# The NEI VFQ-25C: Calibrating Items in the National Eye Institute Visual Function Questionnaire-25 to Enable Comparison of Outcome Measures

**DOI:** 10.1167/tvst.11.5.10

**Published:** 2022-05-11

**Authors:** Judith E. Goldstein, Chris Bradley, Alden L. Gross, Marylou Jackson, Neil Bressler, Robert W. Massof

**Affiliations:** 1 Wilmer Eye Institute, Johns Hopkins University School of Medicine, Baltimore, MD, USA; 2 Department of Epidemiology, Johns Hopkins School of Public Health, Baltimore, MD, USA; 3 Department of Ophthalmology and Visual Sciences, University of British Columbia, Vancouver, BC, Canada

**Keywords:** NEI VFQ-25, patient reported outcome, calibration, Rasch analysis, NEI VFQ

## Abstract

**Purpose:**

To improve the usefulness of the National Eye Institute Visual Function Questionnaire-25 (NEI VFQ-25) by enabling estimation of measures on an invariant scale and comparisons between patients and across studies.

**Methods:**

Datasets of baseline NEI VFQ-25 responses from nine studies (seven retina randomized trials, *n* = 2770; two low vision studies, *n* = 572) were combined. The method of successive dichotomizations was applied to patient ratings of the main NEI VFQ-25 and six supplemental items to estimate Rasch model parameters using the R package ‘msd.’ Calibrated item measures and rating category thresholds were estimated for the NEI VFQ-25, as well as for two domain-specific versions: the NEI VFQ-VF that includes only visual function items and the NEI VFQ-SE that includes only socioemotional items.

**Results:**

Calibrated item measures were estimated from study participants (*n* = 3342) ranging in age from 19 to 103 years, with mean (SD) age of 69.3 (11) years and a mean logMAR visual acuity of 0.30 (Snellen 20/40). Item measure estimates had high precision (standard error range, 0.026–0.085 logit), but person measure estimates had lower precision (standard error range, 0.108–0.499 logit). Items were well targeted to most persons, but not to those with higher levels of function.

**Conclusions:**

Calibrated item measures and rating category thresholds enable researchers and clinicians to estimate visual, socioemotional, and combined measures on an invariant scale using the NEI VFQ-25.

**Translational Relevance:**

Applying NEI VFQ 25C calibrated item measures (software provided) to the NEI VFQ-25, users can estimate overall, visual, and socioemotional function measures for individual patients.

## Introduction

It is now widely accepted that changes in visual acuity, macular thickness, and other clinical observations may not accurately represent intervention outcomes from the patient's viewpoint.^1^^,^^2^ With increasing options for therapies in retinal and other ocular disorders, it is important to include measures of the comparative effectiveness of various interventions from the patient's perspective. Visual function questionnaires (VFQs) provide quantitative estimates of a person's visual function which are dependent on ocular disease state in addition to physical, emotional, and cognitive status. It is the change in these estimates that allow us to measure the outcome of an intervention in units that are meaningful to the patient.

The National Eye Institute Visual Function Questionnaire-25 (NEI VFQ-25) remains one of the most commonly used patient-reported outcome (PRO) measures in ophthalmology studies.^3^^–^^6^ Its intended purpose is to measure both vision- and health-related quality of life. The NEI VFQ-25 is short and simple to administer and provides easy-to-understand scoring instructions that do not require special software or analytic techniques.^7^ The trade-off for simplicity, however, comes with serious instrument-specific and scoring methodology flaws, including multidimensionality (e.g., inclusion of conceptually distinct visual function [VF] and socioemotional [SE] constructs); the use of raw scores that do not satisfy fundamental properties of measurement (e.g., the difference in ability between rank scores 1 and 2 is in general not the same as between 2 and 3); permitting opt-out responses to irrelevant items, which may distort estimates of visual function when using raw scores (e.g., respondents who rate the easy items may erroneously show inflated estimates when using raw scores); problems with item fit validity; and differential item functioning.^8^^–^^12^ The purpose of this work is to resolve many of the aforementioned issues for future use of the NEI VFQ-25. The problem of multidimensionality can only be resolved through domain-specific questionnaires, and we provide calibrated item measures using Rasch analysis for visual function and socioemotional subsets of the NEI VFQ-25, referred to as the NEI VFQ-VF and NEI VFQ-SE, respectively. We also calibrate items for a modified version of the NEI VFQ-25, which we call the NEI VFQ-25C. Although the NEI VFQ-25C does not resolve the issue of multidimensionality, it is important to have an overall NEI VFQ score for two reasons: (1) despite all of the documented psychometric flaws, the NEI VFQ-25 continues to be widely used in research^13^^–^^15^; and (2) there is clinical utility in having an overall score when VF and SE domains depend on the same underlying impairment (e.g., combining anti-vascular endothelial growth factor with cognitive behavior therapy in neovascular macular degeneration).

Like most VFQs, the NEI VFQ-25 contains a set of items (i.e., questions) and asks the patient to rate each item using ordered response options or categories. For example, an item such as “how much difficulty do you have reading street signs or the names of stores” is rated as “no difficulty at all,” “a little difficulty,” “moderate difficulty,” “extreme difficulty,” “stopped doing this because of your eyesight,” or “stopped doing this for other reasons or not interested in doing this.” Except for the general health and general vision questions, the NEI VFQ-25 uses four response category types: “difficulty,” “agreement,” “frequency,” and “severity.” The original item content of the NEI VFQ-25 is based on a focus group of 82 study participants with a broad range of diagnoses and impairments, the majority of whom had visual acuity less than 20/40 in the better eye.^16^^,^^17^ A test version of 52 items was subsequently shortened to the current NEI VFQ-25, which ranges from a total of 26 scorable items to 39 items depending on whether supplemental items are included.

The recommended scoring system for the NEI VFQ-25 generates 12 subscale scores and an overall composite score. The 12 subscales for the NEI VFQ-25 are general health, general vision, ocular pain, near activities, distance activities, social functioning, mental health, role difficulties, dependency, driving, color vision, and peripheral vision. To generate a subscale score, items that were rated within that subscale are first recoded to a 0 to 100 scale and then averaged. The overall composite score is created by averaging 11 of the 12 subscale scores (general health is excluded).^7^ It is worth noting that four of the subscales have only one item (e.g., peripheral vision), and approximately half of the items, including nearly all of the visual function items, have an available opt-out response that is scored as missing data.

Despite its popularity as a patient-reported outcome instrument in ophthalmic clinical research, the recommended scoring system for the NEI VFQ-25 remains widely criticized for its ad hoc design and violations of modern psychometrics.^8^^–^^12^ In particular, the recommended scoring system does not estimate function on a scale whose unit of measurement remains invariant across the scale.^18^^–^^20^ As done with Impact of Vision Impairment^21^ and Activity Inventory,^22^ and intended for the Eye-tem Bank and the Patient Reported Outcomes Measurement Information System,^23^^–^^26^ we apply Rasch analysis to calibrate NEI VFQ items, enabling researchers and clinicians to estimate a single patient or study cohort on an invariant scale. Responses from seven retina treatment trials (primarily macular disease) and two low-vision studies with a total sample size of 3342 were used to calibrate items.^11^^,^^27^^–^^33^ Our approach should benefit clinicians, researchers, and pharmaceutical and medical device companies using the NEI VFQ-25, as well as regulatory bodies such as the Food and Drug Administration that often require PROs to be included among the outcome measures.^34^ Software is provided to facilitate implementation of the calibrated measures.^35^

## Methods

The Johns Hopkins School of Medicine Institutional Review Board determined the project was exempt from review.

### Study Samples and Modifying the NEI VFQ-25

Raw NEI VFQ-25 and appendix item response data from participants in nine studies were pooled for analysis. The studies selected were chosen because they include NEI VFQ-25 administered in the same language (English), and they represent a variety of disorder diagnoses and ranges of visual acuity. Seven datasets (*n* = 2770) from randomized clinical trials of anti-vascular endothelial growth factor therapy for AMD, retinal vaso-occlusive disease, or diabetic retinopathy were joined with two datasets (*n* = 572) from low-vision observational studies representing a mix of disorder diagnoses. Table 1 provides demographic statistics and other information about each dataset. Across all samples, study participants were 18 years or older. Data from each sample included response rank scores at study baseline for each item on the NEI VFQ-25 and six appendix items, visual acuity for each eye, diagnosis, date of birth, and sex. One dataset (LV/MEEI) did not administer the three driving questions and three of the six appendix items.

**Table 1.** Study Participant Characteristics of NEI VFQ-25 Datasets

		Visual Acuity (logMAR)^a^			
Datasets	Female (%)	Mean (SD)	Range	Primary Diagnosis	Age (y), Mean (SD)
ANCHOR^29^ (*n* = 418)	50.00	0.32 (0.36)	−0.30 to 1.60	AMD with CNV	76.50 (7.59)
MARINA^27^ (*n* = 716)	35.20	0.32 (0.30)	−0.30 to 1.30	AMD with CNV	76.50 (6.86)
PIER^28^ (*n* = 184)	40.20	0.29 (0.30)	−0.20 to 1.40	AMD with CNV (or RVO with ME)	78.13 (6.87)
BRAVO^30^ (*n* = 392)	53.10	0.08 (0.21)	−0.30 to 1.20	BRVO with ME	66.23 (11.75)
CRUISE^31^ (*n* = 385)	56.40	0.09 (0.22)	−0.30 to 1.10	RVO with ME	67.36 (12.30)
RIDE^32^ (*n* = 325)	57.20	0.31 (0.26)	−0.20 to 1.30	DM with ME	62.50 (10.18)
RISE^32^ (*n* = 350)	57.40	0.33 (0.26)	−0.20 to 1.10	DM with CSME	62.16 (9.52)
LV/Wilmer^11^ (*n* = 305)	41.70	0.70 (0.57)	−0.12 to 2.85^b^	All LV diagnoses	67.68 (17.05)
LV/MEEI^33^ (*n* = 267)	44.50	0.51 (0.43)	−0.12 to 2.20^b^	All LV diagnoses	67.05 (9.52)

AMD, age-related macular degeneration; CNV, choroidal neovascularization; RVO, retinal vein occlusion; BRVO, branch retinal vein occlusion; ME, macular edema; DM, diabetes mellitus; CSME, clinically significant macular edema; LV (low vision). LV/Wilmer, Low Vision Wilmer Eye Institute; LV/MEEI, Low Vision Massachusetts Eye and Ear Infirmary.

a Represents logMAR of the better eye.

b The logMAR values beyond 1.60 were obtained in LV populations using Berkeley Rudimentary Vision Test charts.

All NEI VFQ-25 items except the first two (overall health and eyesight quality) were included, leaving us with 24 items, plus six supplement items, resulting in a 30-item questionnaire to be used for these analyses. Based on prior published dimensionality analyses, the items were categorized into 20 visual function (of which three are driving related) and 10 socioemotional items (Table 2).^8^^–^^10^ The two items that reference pain—#4 (amount of pain or discomfort in or around your eyes, such as burning, itching, aching, etc.) and #19 (pain or discomfort in or around your eyes keeps you from doing what you'd like)—were categorized into VF and SE, respectively. Consistent with questionnaire scoring recommendations, driving difficulty is rated only from respondents who are currently driving or who have a history of driving, and responses to all three driving questions are considered missing for respondents who never drove or discontinued driving for reasons other than vision or because of eyesight and other reasons (response 2 or 3 to question #15b). For all NEI VFQ-25 items, any response option of “stopped doing this for other reasons or not interested in doing this” was scored as missing data.^7^ The software for implementing our calibrated measures details the transformation of responses from the NEI VFQ-25 to the NEI VFQ-VF, NEI VFQ-SE, and NEI VFQ-25C.^35^

**Table 2.** Item Measures, Standard Errors, and Threshold Estimates for the NEI VFQ-25C, NEI VFQ-VF, and NEI VFQ-SE

			25C		VF		SE	
NEI VFQ-25	Converted	Item	Item	Standard	Item	Standard	Item	Standard
Item No.	Item No.	Description	Measure	Error	Measure	Error	Measure	Error
3	3	How much time worrying about eyesight	1.892	0.031	—	—	2.097	0.033
4	4	Amount of pain or discomfort in or around your eyes	−2.653	0.085	−2.893	0.091	—	—
5	5	Reading ordinary print in newspapers	2.057	0.028	2.368	0.031	—	—
6	6	Doing work up close (e.g., cooking, sewing, fixing things around the house)	0.938	0.029	1.056	0.031	—	—
7	7	Finding something on a crowded shelf	−0.583	0.040	−0.619	0.042	—	—
8	8	Reading street signs or the name of stores	0.467	0.030	0.540	0.032	—	—
9	9	Going down steps, stairs, or curbs in dim light or at night	−0.134	0.039	−0.138	0.042	—	—
10	10	Noticing objects off to the side while you are walking along	−1.113	0.044	−1.189	0.046	—	—
11	11	Seeing how people react to things you say	−1.077	0.037	−1.135	0.039	—	—
12	12	Picking out and matching your own clothes	−2.357	0.054	−2.548	0.057	—	—
13	13	Visiting with people in their homes, at parties, or in restaurants	−1.879	0.042	−2.016	0.044	—	—
14	14	Going out to see movies, plays, or sports events	−0.224	0.028	−0.171	0.029	—	—
15, 15a, 15b, 15c	30	Driving during the daytime in familiar places (relevant to current drivers or those who have discontinued for reasons in part related to vision)	0.104	0.026	0.266	0.028	—	—
16	16	Driving at night	2.527	0.028	3.069	0.033	—	—
16A	16A	Driving in difficult conditions (e.g., bad weather, during rush hour, on the freeway)	1.803	0.026	2.172	0.029	—	—
17	17	Accomplish less than you would like because of vision	0.956	0.027	—	—	0.973	0.030
18	18	Limited in how long you can work or do other activities because of your vision	−0.103	0.029	—	—	−0.277	0.031
19	19	Pain or discomfort in or around your eyes keeps you from doing what you'd like	−2.469	0.052	—	—	−2.990	0.055
20	20	Stay home most of the time because of your eyesight	−0.857	0.031	—	—	−1.170	0.032
21	21	Frustrated a lot because of eyesight	1.701	0.026	—	—	1.829	0.029
22	22	Less control over what you do because of eyesight	1.137	0.026	—	—	1.147	0.028
23	23	Rely too much on what other people tell you because of eyesight	0.010	0.027	—	—	−0.169	0.029
24	24	Need a lot of help from others because of eyesight	−0.113	0.028	—	—	−0.310	0.030
25	25	Worry about doing things that will embarrass you because of your eyesight	−0.821	0.030	—	—	−1.130	0.032
A3/7A	A3/7A	Reading small print in a telephone book or on a medicine bottle or legal forms	2.137	0.029	2.456	0.032	—	—
A4/7B	A4/7B	Figuring out whether bills you receive are accurate	0.408	0.027	0.519	0.029	—	—
A5/7C	A5/7C	Shaving, styling hair, putting on makeup	−1.038	0.038	−1.093	0.040	—	—
A6/10A	A6/10A	Recognizing people you know from across a room because of eyesight	−0.115	0.033	−0.085	0.035	—	—
A7/10B	A7/10B	Taking part in active sports or other outdoor activities (e.g., golf, bowling, jogging) because of eyesight	0.042	0.027	0.122	0.029	—	—
A8/10C	A8/10C	Seeing and enjoying programs on TV	−0.645	0.039	−0.682	0.041	—	—
			Threshold Estimates	Standard Error	Threshold Estimates	Standard Error	Threshold Estimates	Standard Error
			−2.396	0.013	−2.649	0.017	−2.675	0.021
			−0.796	0.010	−1.075	0.013	−0.878	0.016
			0.640	0.009	0.670	0.011	0.190	0.015
			2.187	0.009	2.355	0.011	1.661	0.016

### Rasch Analysis

We employed Rasch analysis to estimate person and item measures (i.e., estimates of person ability and item difficulty) from NEI VFQ-25 participant responses on an invariant logit scale where the difference between *K* and *K* + 1 represents the same difference in visual function for every real number *K*.^8^^,^^36^ With Rasch analysis, missing item responses do not change the measurement scale (Rasch analysis assumes that the underlying latent trait is the same even with a missing response), unlike the recommended scoring strategy of the NEI VFQ-25, where the composite score depends on the number and choice of items rated in each subscale (e.g., rating only easy items changes the raw score). Instead, missing item responses in Rasch analysis change the standard error (i.e., precision of the estimate). A second advantage of Rasch analysis is that a single set of calibrated item measures can be provided for estimating person measures from different studies on the same scale, enabling direct comparisons.^37^ This contrasts with item response theory (IRT), where each item has its own item discrimination parameter, effectively enabling each item to measure persons on its own scale. Item discrimination parameters add mathematical flexibility to IRT models and allow them to model the data better but at the expense of violating a fundamental property of measurement: that all items should measure the latent trait in the same the unit of measurement.^38^ Thus, when the goal is to “measure” something rather than “model” the data, Rasch models are preferred.^39^ Third, Rasch analysis estimates rating category thresholds (boundaries between neighboring rating categories on the real number line) that define the sizes of the intervals representing the rating categories. A very small interval tells us that the rating category is not easy to discriminate from its neighbors, unlike in Likert scales, where every rating category is assumed to be equally discriminable. Fourth, Rasch analysis provides us with standard errors, whereas the NEI VFQ-25 composite scoring strategy cannot. Finally, statistical power is greater when using Rasch analysis instead of composite scores (which under the best of circumstances should be considered nonparametric data).^40^^,^^41^

Rasch analysis has previously been used to estimate item measures, person measures, and rating category thresholds for the NEI VFQ-25.^8^^,^^9^^,^^36^ However, the Rasch models used (e.g., Andrich rating scale model, Masters’ partial credit model) often estimate disordered rating category thresholds, which is inconsistent with the concept of a rating scale, where ordered rating categories are separated by ordered thresholds.^42^^,^^43^ To rectify this problem, advocates of the Andrich and Masters models have recommended merging neighboring rating categories as many times as necessary during post hoc analysis until all estimated rating category thresholds are ordered.^44^ However, this practice creates a rating scale with fewer rating categories than the one administered in the original questionnaire, reducing the responsiveness of the instrument to potential effects of an intervention or exposure. Petrillo and colleagues^9^ pooled six datasets (four of which are represented in this paper) and noted that 15 of the 25 items on the NEI VFQ-25 (plus six supplemental items) show disordered category thresholds when estimated with the partial credit model.^43^ Rather than require post hoc manipulation and modification of the data to estimate ordered thresholds, we used the method of successive dichotomizations (MSD), which is a polytomous response model that always estimates ordered rating category thresholds and has been shown to estimate parameters in near perfect agreement with their true values using simulated rating scale data.^43^^,^^45^

MSD extends the dichotomous Rasch model to multiple rating categories by applying the dichotomous Rasch model to every possible dichotomization of response categories. If the response categories are represented as non-negative integers from 0 to *M*, then MSD applies the dichotomous Rasch model to all *M* - 1 possible dichotomizations: {0} versus {1, 2, …, *M*}; {0, 1} versus {2, 3, …, *M*}; {0, 1, 2} versus {3, 4, …, *M*}; etc. The estimated item and person measures from each of the dichotomizations are then averaged to estimate final MSD item and person measures. The *M* - 1 rating category thresholds are subsequently estimated one threshold at a time. For each dichotomization of response categories, the estimated MSD item and person measures are anchored (i.e., their values are fixed and not estimated), and the remaining parameter in the dichotomous Rasch model (a single threshold) is estimated using maximum likelihood estimation. This mathematical approach toward estimating measures makes MSD a polytomous Rasch model that always estimates ordered rating category thresholds. MSD was implemented using the R package ‘msd.’ Because MSD is applicable only when all items have the same number of rating categories, we required all items in the NEI VFQ-VF, NEI VFQ-SE, and NEI VFQ-25C to have five rating categories.

### Combining Datasets

The primary goals of this study are to improve the psychometric properties of the NEI VFQ-25 response analytics and to provide calibrated item measures on a common scale for the NEI VFQ-VF, NEI VFQ-SE, and NEI VFQ-25C. To achieve these aims, we tested the appropriateness of combining the nine individual studies in Table 1 into a single dataset of 3342 persons by applying a one-way ANOVA to item measures estimated from each of the datasets and from all datasets combined.^46^

## Results

Study participants ranged in age from 19 to 103 years, with mean age of 69.3 years (SD = 11; median = 71 years) as seen in Table 1. Diagnoses related to retinal vaso-occlusive disease and diabetic retinopathy accounted for 43.5% of participants, macular degeneration was present in 39.4%, and a mix of diagnoses causing low vision was present in 17.1%. Better eye best-corrected binocular visual acuity ranged from –0.30 to 2.85 logMAR (Snellen equivalent 20/10 to 20/1416). Mean logMAR was 0.30, and median logMAR was 0.20 (Snellen equivalent values were 20/40 and 20/32, respectively). Females represented 52.1% of the sample.

**Figure 1.**
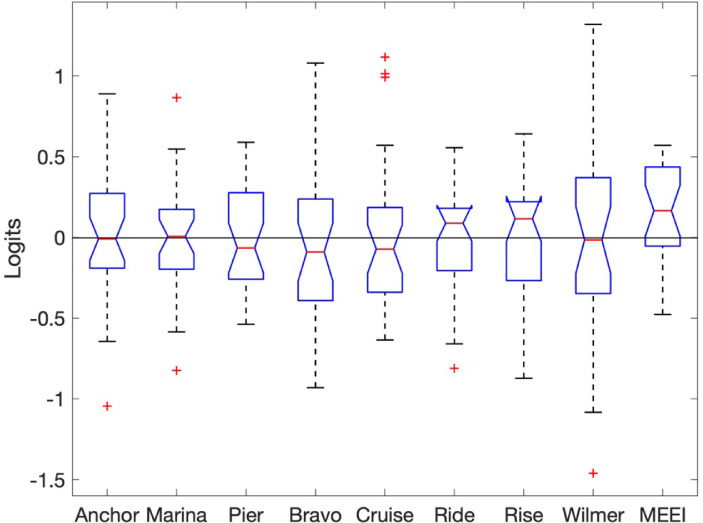
Boxplots of estimated item measures for each study. *Red lines* represent median differences in item measures between each dataset and the combined data. *Boxes* are 25th to 75th percentiles, and *whiskers* include all data points except for the outliers (represented as *plus signs*).

Figure 1 shows boxplots of the difference in item measures estimated for each study and item measures estimated for the combined dataset. An ANOVA testing group–dependent differential item functioning showed no statistically significant difference (*P* = 0.92), suggesting that it was appropriate to combine the nine individual studies in Table 1 into a single dataset of 3342 persons.

Table 2 shows the calibrated item measures and rating category thresholds for the NEI VFQ-VF, NEI VFQ-SE, and NEI VFQ-25C. Item measures in Table 2 demonstrate that different items have different average levels of difficulty for the population of eye patients represented by the nine groups of respondents. We provide Excel programs^35^ (https://sourceforge.net/projects/msd-nei-vfq/files/) for each version that enable users to estimate a single person measure on an invariant scale.

**Figure 2.**
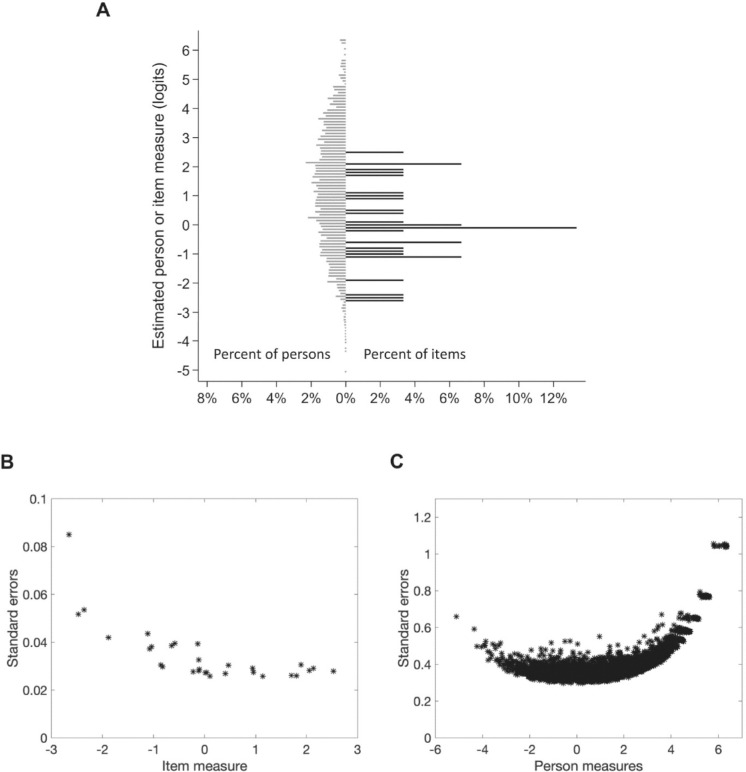
(**A**) Wright construct map for the NEI VFQ-25C. (**B**) Item measure standard errors versus item measures. (**C**) Person measure standard errors versus person measures.

**Figure 3.**
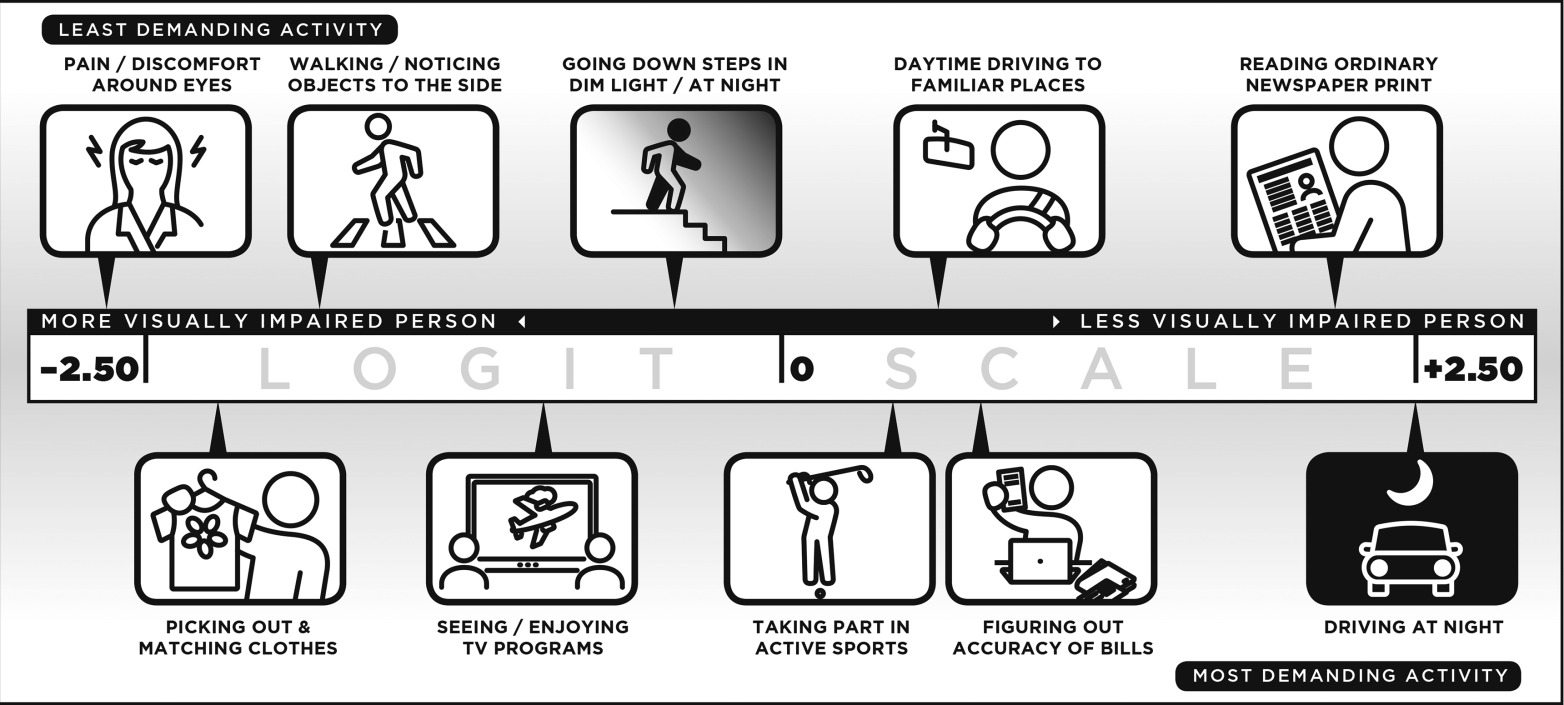
Logit scale with selected items from the NEI-VFQ-25C placed at their estimated item measures. More positive item measures (items to the *right*) require greater visual ability, whereas more negative item measures require less visual ability. When person measures can be placed on the same scale, more positive person measures (persons to the right) have greater visual ability, and more negative person measures have less visual ability.

Figure 2A plots the distributions of estimated item and person measures (Wright construct map) for the NEI VFQ-25C whereby the axis origin is set to the mean item measure (defined to be 0 logit) as seen in Figure 3. The estimated item measures ranged from −2.65 to + 2.53 logits (SD = 1.39 logits), the estimated person measures range from −5.10 to 6.35 logits (mean = 1.26 logits, SD = 1.99 logits), and 28% of the persons were located above the highest estimated item measure of 2.53 logits. In comparison (not shown but data provided in Table 2), person measures for the NEI VFQ-VF ranged from −5.19 to 5.40 logits (mean = 1.17 logits, SD = 1.77 logits), with 15.5% of the persons located above the highest estimated item measure of 3.07 logits. Person measures for the NEI VFQ-SE ranged from −2.99 to + 2.10 logits (SD = 1.56 logits), with 27.6% of the persons located above the highest estimated item measure of 2.10 logits. Thus, the lack of items targeting persons with the greatest function (most positive person measures) is exacerbated when only the NEI VFQ-SE items are queried. Figures 2B and 2C plot the standard errors of the estimate against the estimated item and person measures for the NEI VFQ-25C. The crescent shapes for the standard errors are typical for an unbounded scale (the *x*-axis) and show worse precision for easier items and persons with greatest function. Nevertheless, even for persons well targeted by items at the mean of the measures, the standard errors of the person measure estimates were on average an order of magnitude larger than the standard errors of the item measure estimates (0.50 vs. 0.03); this is primarily due to the small number of items (*n* = 30) and the large number of persons (*N* = 3342), as the standard error of the estimate equals the standard deviation of the measurement uncertainty (test–retest) distribution divided by the square root of the number of observations.

**Figure 4.**
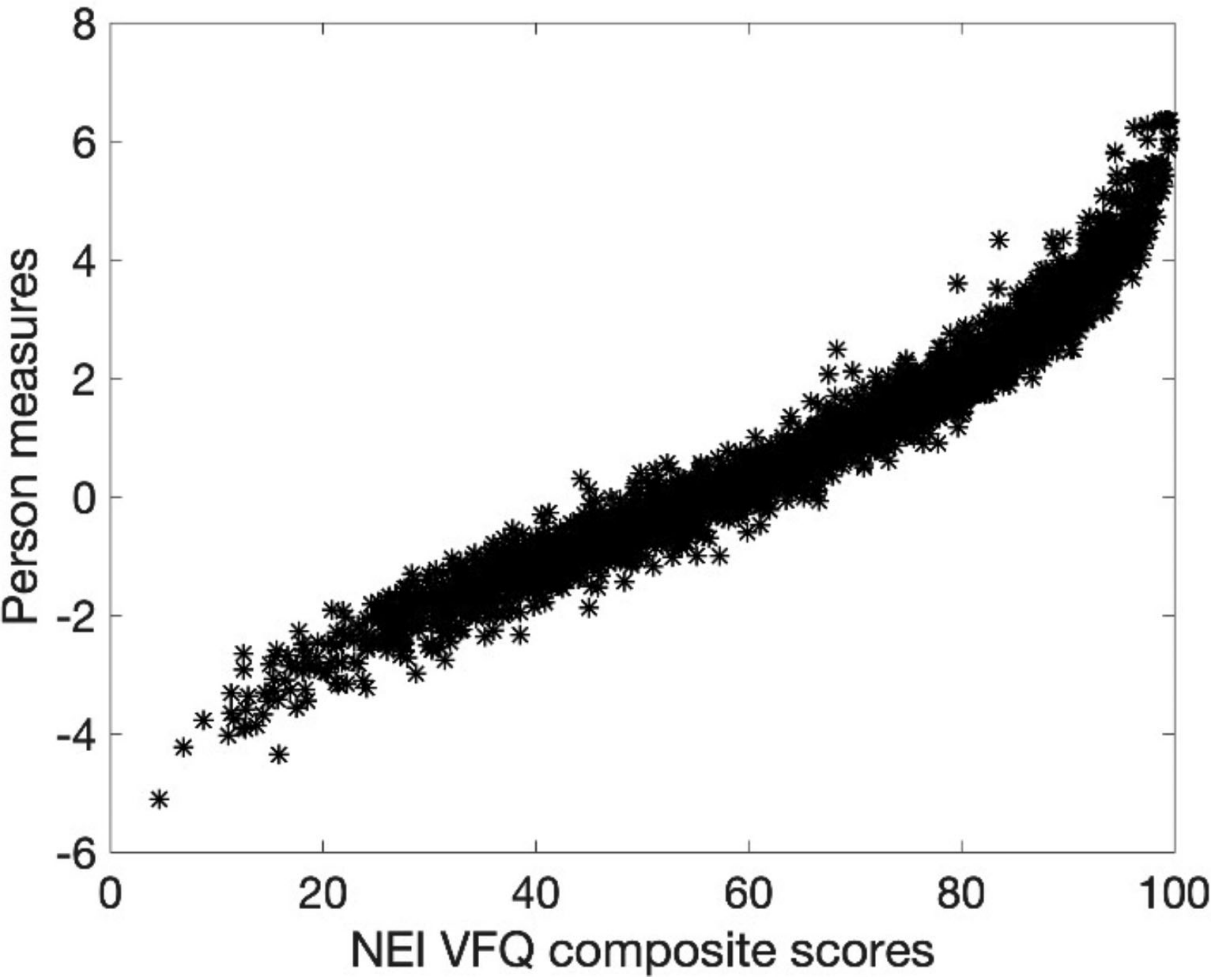
MSD estimated person measures versus NEI VFQ composite scores.

Figure 4 plots estimated person measures against NEI VFQ composite scores. The observed sigmoidal relation and the linear relationship near the origin are consistent with previous observations.^8^^,^^36^ The variance about an expected quasi-logistic function is likely a consequence of distortions from weighted sums of ordinal raw scores and missing data.^11^^,^^36^

## Discussion

In this work, we applied modern psychometric techniques to calibrate items in a modified version of the NEI VFQ-25 called the NEI VFQ-25C and resolved the problem of multidimensionality by providing domain-specific versions: NEI VFQ-VF and NEI VFQ-SE. By calibrating items for a targeted population (in this case, for individuals primarily with retinal disease), researchers and clinicians can now estimate the VF and SE states of study participants and individual patients on an invariant scale. The calibrated item measures for all three measures show excellent precision as evidenced by their small standard errors, whereas the NEI VFQ-VF offers the best targeting. To assist researchers and clinicians who wish to employ the NEI VFQ-VF, NEI VFQ-SE, or NEI VFQ-25C in their research or clinical practice, we have provided three user-friendly Excel programs (macros should be enabled) estimating any individual person measure based on the calibrated item measures and rating category thresholds (https://sourceforge.net/projects/msd-nei-vfq/files/).^35^

As illustrated in the Wright construct map (Fig. 2A) and standard error distributions (Figs. 2B, 2C), the calibrated item measures best discriminate among people with visual function close to the average item measure. The average standard error for the person measures for the NEI VFQ-25C at the point of best discriminability is around 0.4 logit, which means that person measures in this range would have to change by at least 0.78 logit (±0.4 × 1.96 defines the 95% confidence interval) to be statistically significant and thus be scored as exceeding a minimum clinically important difference (MCID), a clinical endpoint.^47^ A major advantage of the proposed approach is that, with traditional NEI VFQ-25 scoring, an MCID cannot be estimated without setting an arbitrary threshold (e.g., 4- or 10-point change), whereas a 95% confidence interval can be estimated with Rasch analysis.^48^^–^^52^ However, when participant function is close to the extremes of the distribution (e.g., monocular loss, early glaucoma or AMD, end-stage disease), the MCID may be as large as 2.0 logits, and clinically relevant changes from baseline function may be difficult to observe with these instruments.^9^^,^^53^ Furthermore, in the extreme case where the study participant responded either with the highest possible rating category to all items or with the lowest possible rating category to all items, the person measure cannot be mathematically estimated and is reported as “NA” by MSD in the provided Excel program. However, our Excel programs provide information in pop-ups regarding the maximum and minimum possible person measures that can be reliably estimated using the instrument (e.g., for the NEI VFQ-25C the maximum possible is 6.6 logit). Thus, any person who responds with only the maximum possible rating will have a person measure larger than 6.6 logit even if it cannot be mathematically estimated.

There are limitations to this study. The calibrations are based primarily on the responses of adult study participants with age-related retinal (primarily macular) disease and relatively good visual acuity and may not generalize to all persons with vision loss, especially persons limited by peripheral vision loss such as those typical of glaucoma and retinitis pigmentosa. Like any patient-reported outcome measure, the item content may be specific to the culture and language of a specific population, and item calibrations reflect a consensus within the targeted population on interpretation of the items.^8^^–^^10^^,^^33^^,^^36^^,^^54^

## Conclusions

The calibrations using Rasch analysis provided for the NEI VFQ-25 enable estimation of visual function (NEI VFQ-VF), socioemotional function (NEI VFQ-SE), and function combining both VF and SE domains (NEI VFQ-25C) for individual patients on an invariant scale. This recommended approach rectifies known problems with composite scores, subscale scores, missing data, and multidimensionality with the NEI VFQ-25.
